# Type I Interferon Gene Response Is Increased in Early and Established Rheumatoid Arthritis and Correlates with Autoantibody Production

**DOI:** 10.3389/fimmu.2017.00285

**Published:** 2017-03-20

**Authors:** Julio E. Castañeda-Delgado, Yadira Bastián-Hernandez, Noe Macias-Segura, David Santiago-Algarra, Jose D. Castillo-Ortiz, Ana L. Alemán-Navarro, Pedro Martínez-Tejada, Leonor Enciso-Moreno, Yolanda Garcia-De Lira, Diana Olguín-Calderón, Leendert A. Trouw, Cesar Ramos-Remus, Jose A. Enciso-Moreno

**Affiliations:** ^1^Medical research Unit of Zacatecas, Mexican Institute of Social Security, UIMZ-IMSS, Zacatecas, Mexico; ^2^National Council of Science and Technology, CONACYT, Catedras-CONACYT, Zacatecas, Mexico; ^3^Departamento de fisiología y farmacología, centro de ciencias básicas, Universidad Autónoma de Aguascalientes, Aguascalientes, Aguascalientes, Mexico; ^4^Unidad de Investigación en Enfermedades Crónico-Degenerativas, Guadalajara, Jalisco, México; ^5^General Hospital: “Emilio Varela Lujan”, Mexican Institute of Social Security, IMSS, Zacatecas, Mexico; ^6^Department of Rheumatology, Leiden University Medical Center, Leiden, Netherlands; ^7^Universidad Autonoma de Guadalajara, Jalisco, México

**Keywords:** rheumatoid arthritis, biomarkers, interferon-1 signature, anticarbamylated protein, anticitrullinated protein antibodies

## Abstract

**Background:**

Rheumatoid arthritis (RA) is an inflammatory debilitating disease that affects the joints in the early and productive phases of an individual’s life. Several cytokines have been linked to the disease pathogenesis and are known to contribute to the inflammatory state characteristic of RA. The participation of type I interferon (IFN) in the pathogenesis of the disease has been already described as well as the identity of the genes that are regulated by this molecule, which are collectively known as the type I IFN signature. These genes have several functions associated with apoptosis, transcriptional regulation, protein degradation, Th2 cell induction, B cell proliferation, etc. This article evaluated the expression of several genes of the IFN signature in different stages of disease and their correlation with the levels of anticitrullinated protein antibodies (ACPA) anticarbamylated protein (Anti-CarP) antibodies.

**Methods:**

Samples from individuals with early and established RA, high-risk individuals (ACPA+ and ACPA−), and healthy controls were recruited at “Unidad de Artritis y Rheumatismo” (Rheumatism and Arthritis Unit) in Guadalajara Jalisco Mexico. Determinations of ACPA were made with Eurodiagnostica ACPA plus kit. Anti-CarP determinations were made according to previously described protocols. RNA was isolated, and purity and integrity were determined according to RNA integrity number >6. Gene expression analysis was made by RT-qPCR using specific primers for mRNAs of the type I IFN signature. Relative gene expression was calculated according to Livak and Schmitgen.

**Results:**

Significant differences in gene expression were identified when comparing the different groups for *MXA* and *MXB* (*P* < 0.05), also when comparing established RA and ACPA− in both IFIT 1 and *G15*. An increased expression of *ISG15* was identified (*P* < 0.05), and a clear tendency toward increase was identified for *HERC5*. *EPSTRI1, IFI6*, and *IFI35* were found to be elevated in the chronic/established RA and early RA (*P* < 0.05). Significant correlations were identified for the IFN signature genes with the levels of ACPA and anti-CarP (*P* < 0.05).

**Conclusion:**

Our data confirm previous observations in the role of IFN signature and the pathogenesis of RA. Also, we provide evidence of an association between several genes of the IFN signature (that regulate Th2 cells and B cell proliferation) with the levels of anti-CarP antibodies and ACPA.

## Introduction

Rheumatoid arthritis (RA) is a chronic, inflammatory, autoimmune disease that affects mainly the diarthrodial joints and is the cause of progressive and incapacitating joint destruction. Systemic manifestations are also present in individuals suffering from the disease, which can be classified as extraarticular manifestations. It is characterized by the presence of inflammatory immune cells in the joints. RA evolves toward a state of synovial hyperplasia, which is the cause of loss of bone structure and functional impairment. Several studies in North America and northern Europe show a prevalence of 0.5–1.1%, whereas in south of Europe, the reported prevalence is of 0.3–0.7%. Few studies have been performed in developing countries using the American College of Rheumatology 1987 criteria (1), showing a prevalence of 0.1–0.5%. However, it is difficult to establish whether this low prevalence is true or represents a referral bias due to the characteristics of healthcare in developing countries (2).

Rheumatoid arthritis affects multiple dimensions of the quality of life of not only patients but also caregivers, such as the physical, emotional, affective and the economy of the family and the health care institutions, all of this accompanied of productivity loss (3). It has been estimated in Mexico that the financial burden of RA on the patient’s families is of 610 ± 302.2 USD/year, whereas the direct costs of RA for the health institutions is of 1,724.00 USD/year/patient generating a great burden in the financial viability of public health care institutions (4). In addition, there is also an increased frequency in the use of biologics such as etanercept, infliximab, adalimumab, and rituximab, increasing significantly the cost of each patient’s treatment.

Several risk factors for RA have been identified, such as allelic variants of *HLA-DR* 01, 04, and others of the shared epitope (5). Also, several genes like the *PAD4* or *CTLA4* have been identified as independent risk factors for the disease (6). Modifiable risk factors such as smoking habits have been associated with severity of RA and with the presence of autoantibodies (7). Other diseases such as periodontal disease and gingivitis associated with several bacterial species such as PAD-like-producing *Porphyromonas gingivalis* have been speculated to increase the availability of citrullinated antigens and therefore the probability of autoantibody generations against such antigens (8). Differences in age for the onset of RA and its symptoms have been proposed to be not only due to the exposure to UV radiation (9) but also due to pesticides, in both the domestic and work environments altering some mechanisms of the oxidative stress response and contributing to tissue damage and RA initiation (10).

The etiology of RA is complex; however, several components of the immune response have been linked to RA physiopathology (11). It has been described the important role of macrophages, dendritic cells, and neutrophils in joint inflammation and synovial destruction in the initial phases of the disease, as the result of type I and II interferons (IFNs) activating myeloid cells and helping to sustain the inflammatory process through the production of cytokines such as IL-6 and TNF-α (12). Synovial fibroblasts also contribute to this process due to increased responsiveness to inflammatory stimuli and reduced apoptosis, perpetuating it (13). Also, this inflammatory milieu and the increased availability of citrullinated and carbamylated antigens lead to the production of autoantibodies (14). Neutrophils contribute to the aforementioned process through the induction of RANK/RANKL activation of osteoclasts, and this has been associated with an increased severity of RA (15). Khandpur et al. have documented that neutrophils of RA patients are more prone to neutrophil extracellular trap formation, and given that these neutrophil-derived structures are rich in citrullinated antigens, they propose that these mechanisms are the ones responsible for autoantibody generation (16).

T cells, both CD4+ and CD8+ are also important actors in the inflammatory response in the synovia, the latter recently linked to increased citrullination through pore-forming mechanisms (17). In a similar fashion, the function of CD4+ Th2 lymphocytes has been suggested to be linked to autoantibody generation and to response polarization through interaction of CD40 and CD40L (18), as well as that mediated by OX-40 and OX40L (19) appear to have a preponderant role in autoantibody production, and these antibodies have an important role in the pathogenesis of disease (20). In the ACR/EULAR2010 criteria, anticitrullinated protein antibodies (ACPA) are considered an important tool to define the disease (21). The increase in ACPA levels is known to precede the onset of symptoms in first-degree relatives of RA patients, and there is also an increased risk of developing RA for the ACPA+ individuals (22). Recently, the presence of anticarbamylated protein (anti-CarP) has also been associated with RA severity and RA in undifferentiated arthralgia (23). This suggests that both ACPA and anti-CarP antibodies might have an important participation in the initiation of symptoms, in the early stages of the disease, and also as markers of tolerance loss (23, 24). Moreover, it has been suggested that in the early stages of RA, type I IFNs might have an important role in the phenomenon of loss of tolerance, and in the chronic or established phase of RA, these molecules contribute to the perpetuation of the inflammatory response (25). Several of the type I IFN genes have been linked to antiviral and immunomodulatory activities (26). Type I IFNs induce dendritic cell maturation and the expression of both co-stimulatory and MHC I/II molecules as well as an increase in cytokine expression (27). Type I IFNs are produced mainly by plasmacytoid dendritic cells in response to a plethora of stimuli such as viral DNA and RNA or due to immune complexes of DNA/RNA antibody (28). IFN-α promotes dendritic cell differentiation and Th0 cell polarization to Th1 and also the cytotoxic response of CD8+ lymphocytes and NKT cells. It has also been described to induce B cell differentiation and antibody production and class switch to IgG (26). In RA, it has been described that immune complexes are produced due to an increased susceptibility to netosis of neutrophils (16), and also autoantibodies could be mediating this process acting as endogenous activators of IFN-α production (29). Although such mechanisms were first described for systemic lupus erythematosus (SLE) (30, 31), recent evidence suggest their participation in other autoimmune diseases such as RA (32). The genes activated through the described mechanism are known as IFN signature and comprise genes such as *MXA* and *MXB* associated with antiviral response (33, 34); *IFIT1* and *IFIT2* linked to posttranscriptional regulation in viral infections (35); *ISG15* and 55 as part of the proteasomal degradation machinery (36); LY6E and RSAD2 are involved in metabolism and proliferation regulation of B cells (37); also, *EPSTRI1*, IFI44L, and *IFI35* (38) are thought to participate in IFN signature response modulation. *IFI6* has been described to participate in apoptosis inhibition mediated by TRAIL (39).

In other autoimmune diseases such as SLE, the immune complexes have been described to be internalized by pDC through the Fc-γRIIA into endosomes and interact with TLR7 and TLR9. After this activation, MyD88 induces activation and phosphorylation and translocation to the nucleus of the IFN-regulating factor 7 nuclear factor, inducing the transcription of the type I IFN genes (40).

Van der Pouw Kraan and colleagues identified several changes in gene expression in the IFN signature in established RA compared to control individuals (25), but the early stages of RA [early RA (eRA)] or the preclinical autoimmune phase of the disease was not the focus of that publication, and there are no available data regarding the role of the induced genes in these preclinical or early stages of the disease. Because of the direct association of several genes of the IFN signature with the physiopathology of RA, the natural history of the disease, and the expansion, regulation, and function of B cells, it is important to understand the relationship of the IFN signature activation and gene expression with the levels of ACPA and anti-CarP. The aims of this article were to analyze whether type I IFN-regulated genes were underexpressed or overexpressed in several groups of individuals at high risk of developing RA and in people with early or established disease and also to evaluate their relationship with autoantibodies in serum.

## Materials and Methods

### Patients

The study participants were recruited between 2012 and 2014 at a Rheumatology Clinic in Guadalajara, Jalisco, Mexico, and at the “Emilio Varela Lujan” General Hospital of Zacatecas, Mexican Institute of Social Security (IMSS). Patients with eRA <2 years of symptoms without treatment (41) and chronic/established RA (cRA) with an evolution of >2 years and treated with disease-modifying antirheumatic drugs (DMARD) were recruited. All study participants were evaluated by board-certified rheumatologists, and diagnosis was confirmed or ruled out according to the 2010 revised European League Against Rheumatism/ACR criteria (42). Informed consent was obtained from all participants. This consent was approved by the local Ethics Committee (IMSS; R-2013-785-009). First-degree relatives of patients with confirmed RA were invited to participate in the study and were categorized into two groups: those negative (ACPA−) and positive (ACPA+) for ACPA. Unrelated healthy control (HC) subjects without self-reported history of autoimmune diseases, malignancy, or infectious diseases (HIV and HCV) were also enrolled. Blood samples were drawn for all subjects, and RNA later (Invitrogen) was used for RNA stabilization. Blood samples treated with RNA later and serum samples were stored at −20°C until use.

### RNA Extraction, Quality Control, and cDNA Synthesis

RNA isolation was performed according to a QIAamp protocol (Qiagen, Germany) adapted in our laboratory. Briefly whole blood samples that had been previously stabilized were unfrozen at room temperature and mixed vigorously in a 15-ml conical tube, and chloroform was added to the sample and subjected to centrifugation. The aqueous phase was transferred to another tube, and precipitation of RNA was made by means of ethanol. The mix was passed through a QIA-shredder spin column, and samples were processed as recommended by the supplier by another step of column purification and RNAse-free DNA digestion in column. Elution of the purified RNA was made in RNAse-free, diethyl pyrocarbonate (Sigma, St. Louis)-treated water. RNA integrity was assessed with the Bionalyzer 2100 (Agilent technologies, USA), and samples with RNA integrity number >6 were used. The conversion of RNA to cDNA was made by means of the Superscript II reverse transcriptase kit (Invitrogen, USA), briefly, 2.5 μg of total RNA was retrotranscribed according to the manufacturers protocol in a Biometra Gradient model T thermal cycler (Thermo Corp., Germany). cDNA was treated with DNAse-free RNAse H, and aliquots of cDNA at a concentration of 25 ng/μl were prepared for the qPCR assays.

### Gene Expression Analysis

Oligonucleotides and probes used in the qPCR assays were designed in the ROCHE Universal Probe Library. All assays were carried out in a Lightcycler 480 (ROCHE, USA) and with the light cycler Taqman Master kit (Roche, USA) with a cycling program as follows: preincubation 95°C/10 min, 45 cycles (denaturation 95°C/10s, Hyb 65°C/30s, and extension 70°C/1s), and cooling to 40°C. From the amplification curves, Ct was obtained for each sample, for both the problem genes (Table 1; IFN signature genes) and the constitutive gene hypoxanthine phosphoribosyltransferase. Gene expression analysis was calculated with the 2−ΔΔCt using the HC group as a reference.

**Table 1:** **Primer sequences for qPCR**.

Gene name/symbol	Forward	Reverse	Accession number
*IFIT1*	gat gta tta cca cat ggg cag a	tag cgg aag gga ttt gaa ag	NM_001548.3
*IFIT2*	atc ccc cat cgc tta tct ct	cca cct caa tta atc agg cac t	NM_001547.4
*IFI44L*	tgc taa gga gta tag cag atg acc ta	cca caa cat cac tct cac ttt aag a	NM_006820.2
*ISG15*	gag gca gcg aac tca tct tt	agc atc ttc acc gtc agg tc	NM_005101.3
*MXA*	atc cag cca cca ttc caa	caa caa gtt aaa tgg tat cac aga gc	NM_002462.3
*MXB*	ttc ttc aaa cac atc cat att tca	cag tgg taa gtc ttt ctg cca gt	NM_002463.1
*EPSTRLI1*	ccg gag aaa tga gat aca aag aat	ggt gaa ccg gtt tag ctc tg	NM_001002264.1
*RSAD*	atg tga aag ccc aag gac ac	ttt ggt ttc aaa taa cac tga ttg a	NM_080657.4
*HERC5*	ctt cca gtg aaa gta tca tca agt g	cca gag caa aat gct ttg att	NM_016323.2
*Ly6E*	atc ttc ttg cca gtg ctg ct	gct tca gga agt aca gat tgc	NM_002346.2
*IFI6*	tgc ttc tct tct ctc ctc caa	gct ctc cga gca ctt ttt ctt	NM_002038.3
*IFI35*	caa aag gag cac acg atc aa	act caa ctg gct gga cat cat	NM_005533.4
Hypoxanthine phosphoribosyltransferase	tga cct tga ttt att ttg cat acc	cga gca aga cgt tca gtc ct	NM-000194.2

*All assays were designed in the Universal Probe Library assay design center (ROCHE), and *in silico* tests were run for each pair of primers using the primer BLAST platform at NCBI*.

### ACPA and Anti-CarP Antibody Determinations

Serum samples collected from the participants were used for autoantibody (ACPA and Anti-CarP antibodies) measurements. Determination of anti-CCP2 antibodies was performed using enzyme-linked immunosorbent assays according to the manufacturer’s instructions (Euro-Diagnostica, Malmö, Sweden). The cutoff value for positivity was set at 25 arbitrary units/ml according to the suggestion of the manufacturer. Determination of anti-CarP was made as described before (43). The plates were incubated with carbamylated or uncarbamylated fetal calf serum (half plate each), washed with PBS/0.05% Tween (Sigma, USA), and subsequently blocked for 6 h at 4°C with 100 μl of PBS/1% BSA (Sigma, USA). After washing, the wells were incubated with 50 μl serum 1/50 diluted in PBS/1% BSA/0.05% Tween. Antibodies were detected using horseradish peroxidase (HRP)-conjugated rabbit anti-human Ig antibody (ABCAM, UK), and HRP-conjugated HRP enzyme activity was visualized using ABTS (PeproTech, UK). As a standard, serial dilutions of a pooled serum sample from a patient with RA were used.

### Statistical Analysis

Normality of data was assessed by a D’Agostino-Pearson normality test. According to data distribution, the multiple comparisons tests were performed: Kruskal–Wallis test with Dunn post hoc test was used for non-normally distributed data and the parametric one-way ANOVA with Tukey post hoc test, for the normally distributed data. Correlation analysis was performed by means of a Spearman correlation. Level of significance was established at α = 0.05. Graph pad prism 5.0 (Graph Pad Software, USA) and SPSS v. 18 (Microsoft, USA) were used for the analysis of the data.

## Results

The analysis of gene expression of the type I IFN pathway has been associated with increased inflammatory responses in other autoimmune disease such as SLE. There is limited information regarding the role of the transcriptional pathway and the relationship that this might have with the inflammatory response in RA in the early stages of the disease and in the high-risk ACPA+ individuals. Therefore, we evaluated the gene expression of several genes associated with the IFN signature in the Mexican population. Given that several genes regulated by type I IFN can be grouped into several functions, such differences are described below. No differences in gender proportions were identified, and all patients belonged to a Mexican Mestizo population, which was not characterized for the presence of HLA-risk Alleles. All patients were treated (in case of RA patients) with DMARD, and methotrexate was used most frequently.

### Antiviral Response Genes of the IFN Signature Are Overexpressed in RA

**Figure 1:**
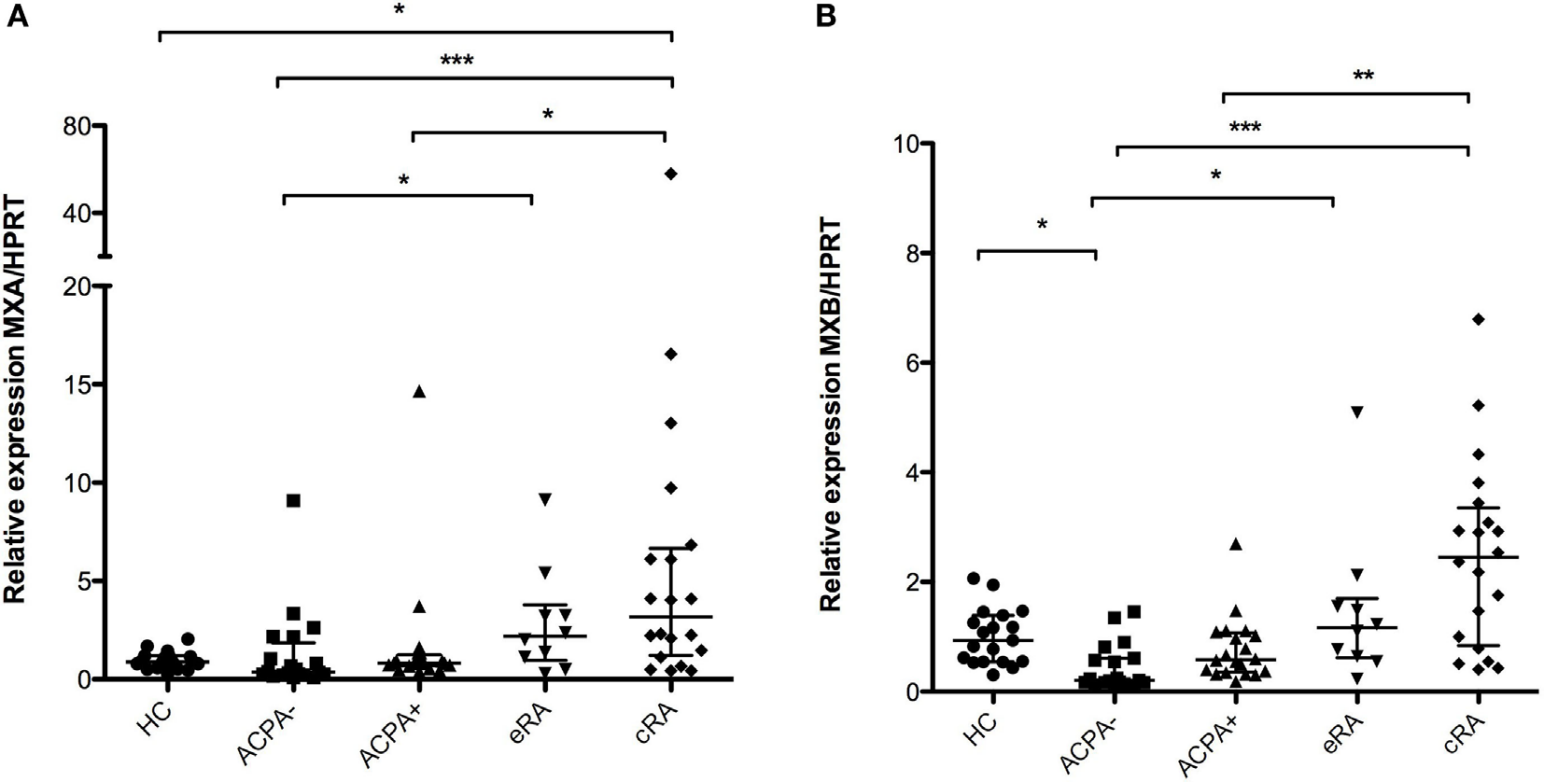
**Analysis of gene expression associated with antiviral response**. Gene expression analysis was carried out in cDNA synthesized from blood total RNA to assess the relative gene expression profile of type I interferon-related genes: **(A)** *MXA* and **(B)** *MXB* genes were analyzed. HC, healthy controls (*n* = 20); ACPA−, first-degree relatives of rheumatoid arthritis (RA) patients negative to anticitrullinated peptide antibodies (*n* = 20); ACPA+, first-degree relatives of RA patients positive to anticitrullinated peptide antibodies (*n* = 20); eRA, early RA patients (*n* = 10); cRA, chronic RA patients (*n* = 20). The graphs depict median ± IQR as descriptive statistics. Multiple comparisons tests were made by means of the non-parametric Kruskal–Wallis test. *P* values of less than 0.05 were considered statistically significant (*P < 0.05; **P < 0.01; ***P < 0.001).

An increased expression of the *MXA* mRNA was found when comparing the established RA (cRA) group with the high-risk group (ACPA+) and also with the control groups (both ACPA− and HC) as shown in Figure 1A (*P* < 0.05). No differences in gene expression were found for the eRA group in any comparison for this gene. *MXB*, another gene, involved in the antiviral response mediated by the type I IFN response was also found to have an increased expression when compared with the control groups (HC and ACPA−) and with the ACPA+ group (Figure 1B, *P* < 0.05), and the same pattern of increase across groups is observed as in *MXA*.

### Transcriptional Regulators of IFN Signature Are Overexpressed in Established RA

**Figure 2:**
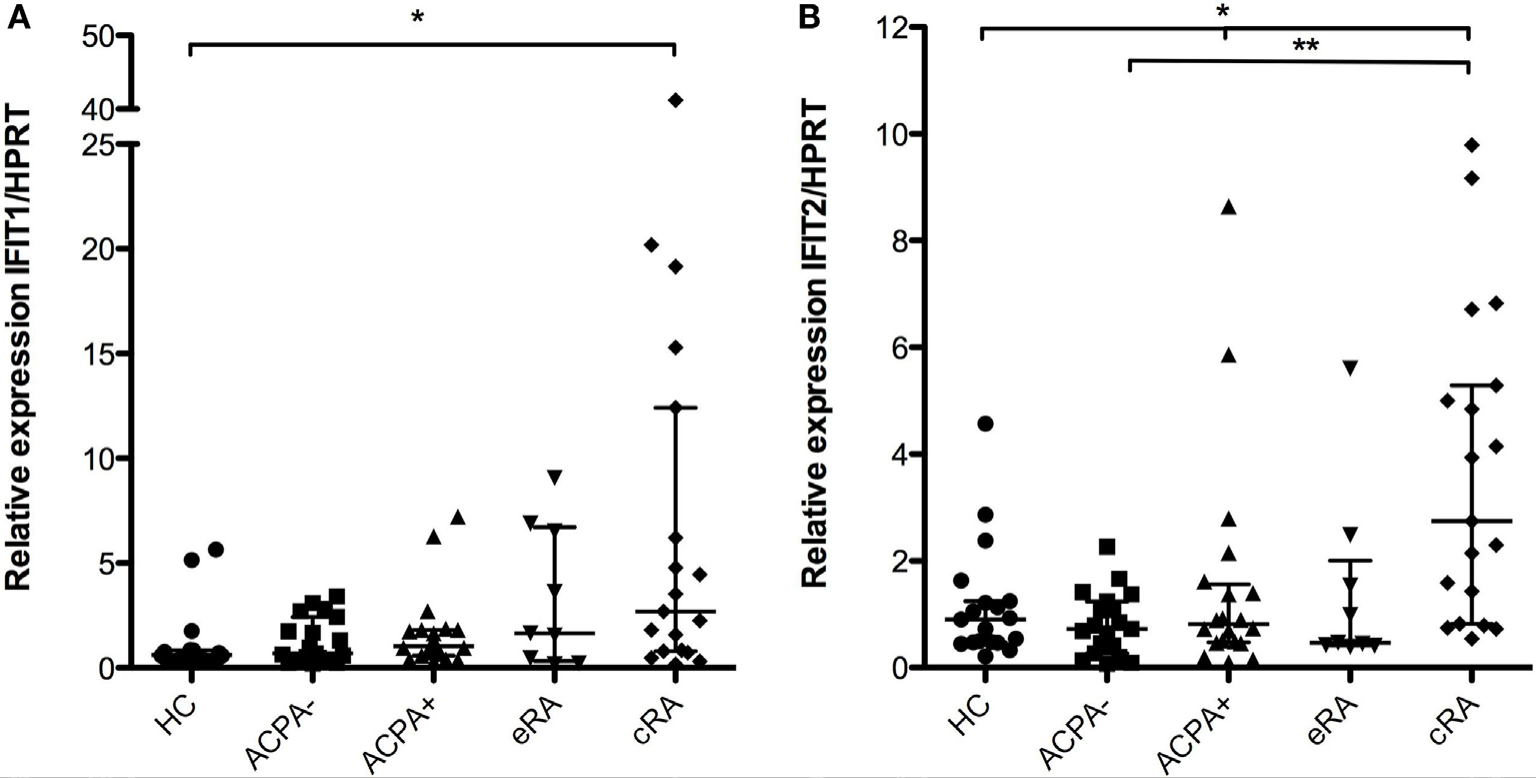
**Gene expression of the posttranscriptional regulators *IFIT1* and *IFIT2***. Gene expression analysis was carried out in cDNA synthesized from blood total RNA to assess the relative gene expression profile of type I interferon-related genes: **(A)** *IFIT1* and **(B)** *IFIT2* genes were analyzed. HC, healthy controls (*n* = 20); ACPA−, first-degree relatives of rheumatoid arthritis (RA) patients negative to anticitrullinated peptide antibodies (*n* = 20); ACPA+, first-degree relatives of RA patients positive to anticitrullinated peptide antibodies (*n* = 20); eRA, early RA patients (*n* = 10); cRA, chronic RA patients (*n* = 20). The graphs depict median ± IQR as descriptive statistics. Multiple comparisons tests were made by means of the non-parametric Kruskal–Wallis test. *P* values of less than 0.05 were considered statistically significant (*P < 0.05; **P < 0.01; ***P < 0.001).

*IFIT1* gene expression was evaluated in the samples of the previously described groups. Significant differences were identified when comparing cRA with ACPA− individuals (Figure 2A, *P* < 0.05). Similar observations can be made for the *IFIT2* gene that belongs to the same family of transcriptional regulators; significant differences were identified for this gene between the cRA and both the ACPA and HC groups (Figure 2B, *P* < 0.05).

### Overexpression in Established RA of the Proteasomal Degradation Machinery of the IFN Signature

**Figure 3:**
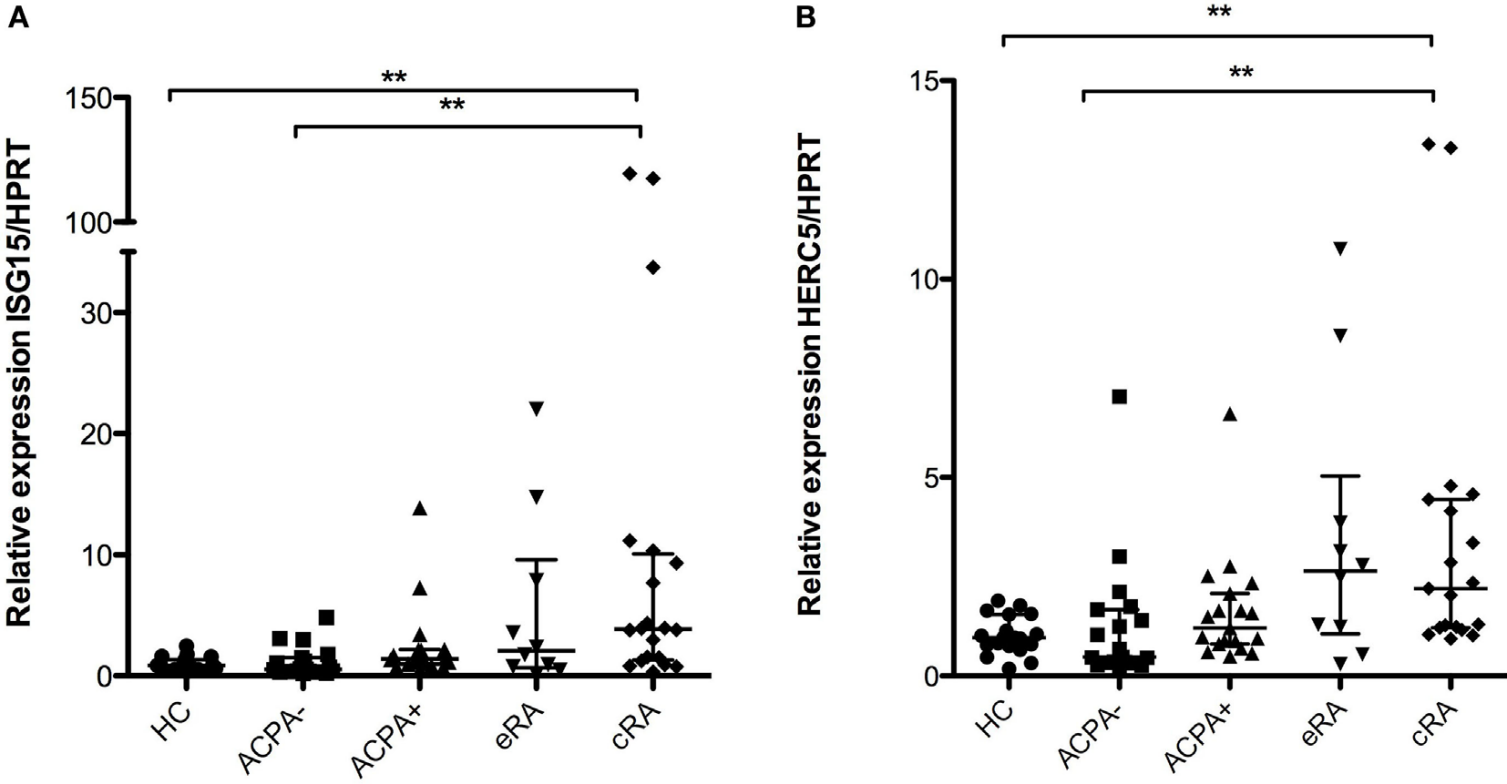
**Interferon (IFN)-1 signature genes associated with induction of proteasomal degradation**. Gene expression analysis was carried out in cDNA synthesized from blood total RNA to assess the relative gene expression profile of type I IFN-related genes: **(A)** *ISG15* and **(B)** *HERC5* genes were analyzed. HC, healthy controls (*n* = 20); ACPA−, first-degree relatives of rheumatoid arthritis (RA) patients negative to anticitrullinated peptide antibodies (*n* = 20); ACPA+, first-degree relatives of RA patients positive to anticitrullinated peptide antibodies (*n* = 20); eRA, early RA patients (*n* = 10); cRA, chronic RA patients (*n* = 20). The graphs depict median ± IQR as descriptive statistics. Multiple comparisons tests were made by means of the non-parametric Kruskal–Wallis test. *P* values of less than 0.05 were considered statistically significant (*P < 0.05; **P < 0.01; ***P < 0.001).

Regulation of protein degradation is a hallmark of the type I IFN signature, and given their role in antigen presentation by class I molecules of due to cross-priming, the expression of the “ISGlation” machinery of proteasomal degradation was also evaluated. Significant differences in gene expression were found for both the *ISG15* and the *HERC5* mRNAs when comparing the established disease patients with HC (Figures 3A,B, respectively). Also, a tendency toward the increase is observed in the natural history of the disease.

### Increased Expression of Proliferation Regulators of the IFN Signature in RA Patients

**Figure 4:**
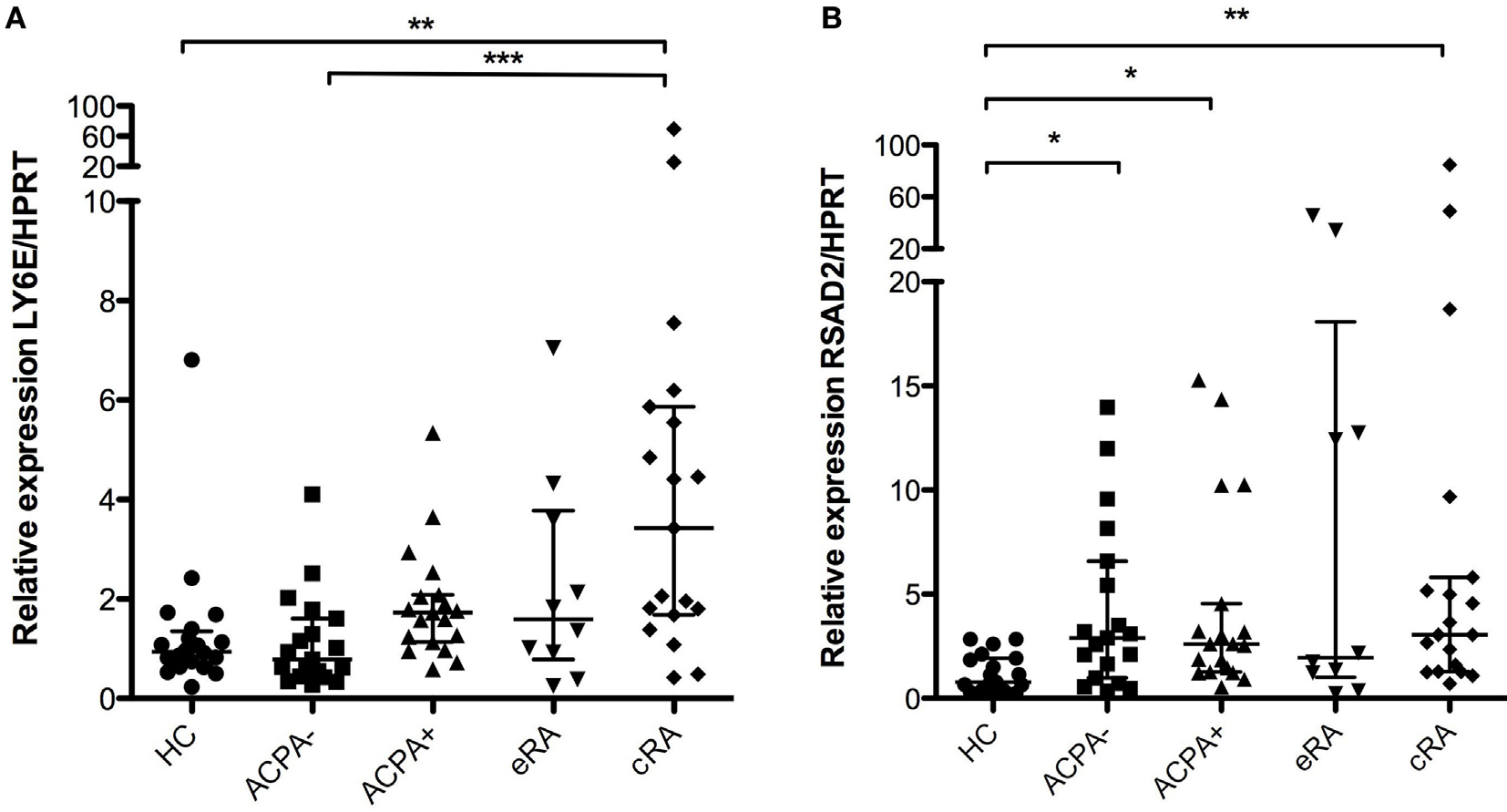
**Analysis of gene expression of the interferon (IFN)-1 signature associated with increased proliferation**. Gene expression analysis was carried out in cDNA synthesized from blood total RNA to assess the relative gene expression profile of type I IFN-related genes: **(A)** Ly6E and **(B)** RSAD2 genes were analyzed. HC, healthy controls (*n* = 20); ACPA−, first-degree relatives of rheumatoid arthritis (RA) patients negative to anticitrullinated peptide antibodies (*n* = 20); ACPA+, first-degree relatives of RA patients positive to anticitrullinated peptide antibodies (*n* = 20); eRA, early RA patients (*n* = 10); cRA, chronic RA patients (*n* = 20). The graphs depict median ± IQR as descriptive statistics. Multiple comparisons tests were made by means of the non-parametric Kruskal–Wallis test. *P* values of less than 0.05 were considered statistically significant (*P < 0.05; **P < 0.01; ***P < 0.001).

Proliferation and expansion of B cells is of major importance in inflammatory autoimmune diseases due to the generation of pathogenic antibodies. Given that several genes of the IFN signature pathway are associated with the regulation of proliferation in B cells, we sought to evaluate the expression of such genes in this phenomenon. Ly6E expression showed significant differences between established RA (cRA) and the ACPA− first-degree relatives and HC as shown in Figure 4A (*P* < 0.05). Also, RSAD2 has been shown to have similar functions, participating in the control of expansion of several immune cells (44). Significant differences were identified when comparing not only established RA (cRA) with HC but also the ACPA+ with HC, suggesting their involvement in early preclinical features of the disease and in the established inflammatory milieu of arthritis (Figure 4B, *P* < 0.05).

### *IFI6, IFI35*, and *EPSTRI1* Gene Expression Is Increased in RA

**Figure 5:**
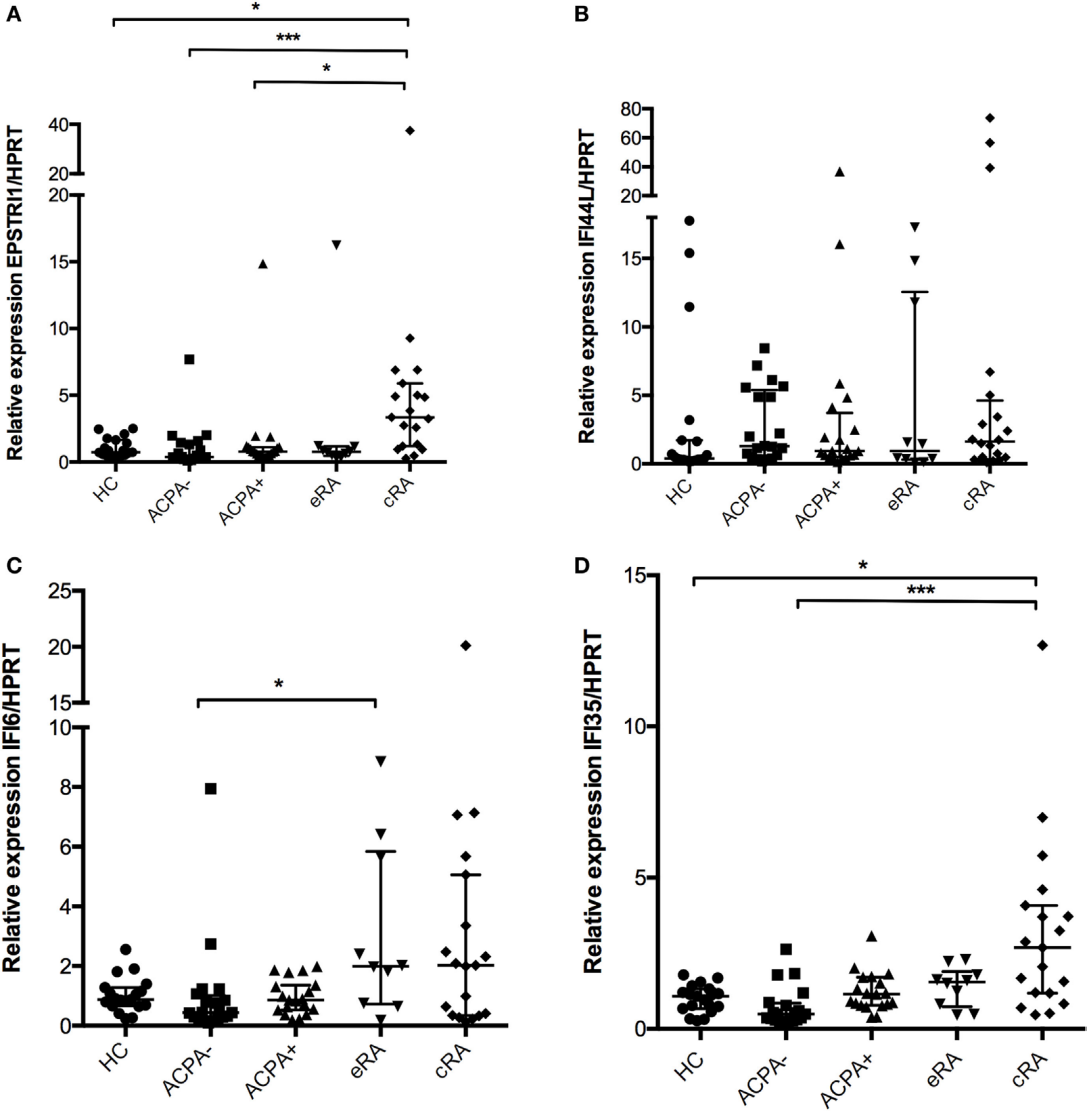
**Genes associated with miscellaneous functions in the interferon (IFN)-1 signature**. Gene expression analysis was carried out in cDNA synthesized from blood total RNA to assess the relative gene expression profile of type I IFN-related genes: **(A)** EPSTRLI1, **(B)** IFI44L, **(C)** *IFI6*, and **(D)** *IFI35* genes were analyzed. HC, healthy controls (*n* = 20); ACPA−, first-degree relatives of rheumatoid arthritis (RA) patients negative to anticitrullinated peptide antibodies (*n* = 20); ACPA+, first-degree relatives of RA patients positive to anticitrullinated peptide antibodies (*n* = 20); eRA, early RA patients (*n* = 10); cRA, chronic RA patients (*n* = 20). The graphs depict median ± IQR as descriptive statistics. Multiple comparisons tests were made by means of the non-parametric Kruskal–Wallis test. *P* values of less than 0.05 were considered statistically significant (*P < 0.05; **P < 0.01; ***P < 0.001).

Other genes of the IFN signature such as *IFI6* have been associated with apoptosis regulation (*IFI6* and *IFI35*), epithelial to mesenchymal transition (*EPSTRI1*), and other functions. There is an increase in the *EPSTRI1* gene expression in the cRA group compared to HC (Figure 5A, *P* < 0.05). No differences were found for IFI44L gene expression for any of the groups analyzed (Figure 5B, *P* > 0.05). A significant higher expression of *IFI6* was observed in the eRA group compared to ACPA− individuals and HC (Figure 5C, *P* < 0.05). *IFI35* also showed significant differences in the cRA group when compared to HC (Figure 5D, *P* < 0.05).

### Autoantibodies against Carbamylated and Citrullinated Proteins Correlate with the Expression Levels of Several Type I IFN-Regulated Genes

Given the importance of several of the abovementioned genes in the regulation of proliferation of immune cells, as well as in the response in the antiviral response and (indirectly) in antigen presentation and consequently in the generation of antibodies by differentiation of B cells into plasmatic antibody-secreting cells, we wondered whether autoantibody levels might be associated with the gene expression of the IFN signature. After a non-parametric Spearman correlation analysis, we identified significant correlations with the levels of anti-CarP antibodies for *IFI6, IFI35, ISG15*, Ly6E, and *MXA* (Spearman *r*, 0.249–0.352, *P* < 0.05; Table 2). In addition, significant correlations were identified for 8 of the 12 IFN signature genes (*IFI6, IFIT2, IFI35, ISG15, MXB*, Ly6E, *EPSTRI1*, and *MXA*; Spearman *r*, 0.227–0.476, *P* < 0.05), suggesting an important participation of the gene regulation mediated by the IFN signature in autoantibody generation. Also a trend for the increase of both anti-CarP and ACPA is observed when comparing for the natural history of the disease (data not shown).

**Table 2:** **Correlation analyses of autoantibody levels [anticitrullinated protein antibodies (ACPA) and anticarbamylated protein (anti-carP) antibodies] with relative expression of interferon-1 signature genes**.

	Relative expression											

	IFI44L	*IFI6*	*IFIT1*	*IFIT2*	*IFI35*	*ISG15*	*MXB*	Ly6E	RSAD2	*HERC5*	*EPSTRI1*	*MXA*
Anti-CarP	0.111	0.249*	0.187	0.101	0.288*	0.294*	0.226	0.264*	0.161	0.197	0.125	0.352**
	0.383	0.047	0.139	0.425	0.021	0.018	0.072	0.035	0.203	0.118	0.327	0.004
	64	64	64	64	64	64	64	64	64	64	64	64
ACPA	0.33	0.261*	0.146	0.417***	0.438***	0.476***	0.273**	0.227*	−0.121	0.188	0.444***	0.287**
	0.750	0.011	0.158	0.000	0.000	0.000	0.007	0.027	0.241	0.067	0.000	0.005
	95	95	95	95	95	95	95	95	95	95	95	95

*Data are presented as follows: correlation coefficient, two-tailed P value, and N*.

*Spearman correlation analysis was carried out*.

P values of <0.05 were considered significant (*P < 0.05; **P < 0.01; ***P < 0.001).

*D’Agostino-Pearson normality test was carried out for the assumption of normality of the data*.

## Discussion

Rheumatoid arthritis is an inflammatory disease that is characterized by the generation of autoreactive clones of T and B cells and, in consequence, by the generation of autoantibodies (45). It has been proposed that autoantibodies could be involved in the generation of IFN through the activation of the pDC, and it could be implicated in the perpetuation of the inflammatory process as has been previously described for LES (46, 47). The first report that linked the IFN signature with autoimmunity in RA came from Van der Paw; he found an elevation in the expression pattern of several of the genes of the pathway in the established disease (25). However, little has been done to try to replicate such data in other populations and settings. Here, we report the analysis of several genes of the type I IFN signature and its relationship with high-risk individuals (ACPA+), eRA, and established RA and the participation of such genes in the physiopathology of the disease in different stages and their possible use as biomarkers in early stages of disease. Also, we discuss the association between the presence of anti-CarP and ACPA and the potential role of the type I IFN signature genes in the generation of such antibodies.

It has been reported that some chronic viral infections might be the trigger or at least are associated with, of autoimmunity, in particular due to cross-reactivity, as has been observed in several tropical viral infections such as Chikungunya (48), Epstein-Barr (49), and Zika (50), which have been associated with acute arthritis of unknown origin and have been poorly characterized (51, 52). In this regard, several genes of the IFN signature such as *MXA* and *MXB* have been associated with protection against viral infection (53, 54). Our data clearly show an increased expression of the *MXA* and *MXB* genes providing evidence of the role that these molecules might have in the induction of inflammation in the early phases of RA or even previous to the onset of symptoms in individuals at high risk, with the possible involvement of such molecules even in the preclinical autoimmune phase of RA, explaining just in part the lack of differences in the established phase of the disease. The risk of developing RA associated with increased expression of these genes needs to be further evaluated in the light of the induction of tolerance processes and determine whether *MXA* and *MXB* gene expression increase is due to viral infection and/or the generation of interferogenic complexes.

Recently, a strong association between RA and the unfolded protein response (UPR) was described (55–57), and therefore, the regulation of the transcription/translation process has been put on the spotlight of RA pathogenesis. It has been described that several regulators of the translation of proteins particularly *GADD34* are associated with the UPR in RA (55); however, little attention has been put to the transcriptional process and their regulators such as *IFIT1* and *IFIT2* that are known to regulate the apoptotic process (58). In this regard, we found an increased expression of these genes in the established disease phase. Suggesting that the role of the transcriptional regulation in the chronic phase of RA might be associated with the activation of the immune response by type I IFN; also, given that due to medication several viral infections become recurrent in these patients (59), upregulation of *IFIT1* and *IFIT2* could be associated with these infections in RA. Also the role of other regulators in the expression of these genes such as miRNA needs to be further evaluated, and also the viral subclinical infection status of such individuals (Epstein-Barr virus and cytomegalovirus infection status) needs to be determined.

The mechanisms of ubiquitination are very important for the clearance of several proteins within the cell and therefore are related to the UPR (60). *ISG15* is a small ubiquitin molecule associated with antiviral response in a process called “ISGlation” (61). It has been described that this molecule can regulate the activity of negative regulator IRF3 and in this way promotes the expression of genes of the IFN signature (62). Further research on the role of *HERC5* and *ISG15* in RA pathogenesis is needed to establish their participation in experimental models of RA and to evaluate how is regulated in the early phases of disease or if the phenomenon is exclusively associated with chronic inflammatory processes as observed in the established disease patients. Also, the potential use of these molecules as markers of treatment success has been described previously by Raterman and colleagues (63).

The regulation of apoptosis may also play an important role in the pathogenesis of RA (13). Little is known about the function of *IFI6*, and it has been described that *IFI6* inhibits apoptosis trough the downregulation of caspase 3 and casase 9 (64). This effect might be associated with increased survival of inflammatory cells that perpetuate the activation of antibody-producing B cells, and this needs to be further evaluated. Also, Ly6E has been associated with an increased proliferation in several cell lineages (65) and could mediate the process of B cell expansion in inflammatory disorders through a TGF-beta-mediated mechanism, but this needs to be further explored. RSAD2 (also known as Viperin) is an antiviral protein associated with regulation of proliferation of immune cells, and it has been associated with Th2 polarization of immune cells through a mechanism mediated by GATA3, NF-κB, and IL-4 production, therefore inducing and modulating the production of autoantibodies (44). The increased expression of RSAD2 in ACPA+ individuals and cRA patients might contribute to autoantibody generation. The increased expression of this pathway needs to be further elucidated given that expression of these molecules is mainly regulated by viral infection (66–68).

There is no clear evidence if the perpetuation of the inflammatory process through the activation of inflammatory cells might be associated with increased levels of antibodies in several stages of the disease (69, 70). For this purpose, we analyzed the expression of several genes of the type IFN gene signature in groups of individuals who had no previous family history of the disease, HC, and also in individuals with ACPA−, ACPA+, eRA, and cRA, which accounts for the natural history of the disease. We found a significant correlation between the expression of these genes associated with proliferation control of B cell maturation and antibody production such as *IFI6, IFI35, ISG15, EPSTRI1, MXA*, and Ly6E, several of which are associated with promotion of B cell differentiation and antibody production (71). Our results differ from those of other groups in which no association has been found for autoantibody production of ACPA and other autoantibodies (RF, antinucleosome, etc.) in adalimumab-treated patients (72), which has been recently confirmed to modify the gene expression pattern of the type I IFN signature in neutrophils of patients with RA (73). Also the authors did not analyze the correlation effect of the IFN signature gene expression on the antibodies that were analyzed, neither did they analyze the anti-CarP antibodies. Another substantial difference that may account for the differences in our findings and that of *Cantaert* et al is that we included individuals (eRA) who were virgin to treatment (72). Our groups of individuals were recruited in an attempt to emulate the natural history of disease in a patient with RA, and therefore, the tendency is clear to increase the levels of autoantibodies in the late phases of disease. Ethnic and population differences may also account for such differences (74, 75).

Although our correlation analysis cannot be confirmed as causality, it clearly highlights the association of the increased expression of Type I IFN response genes with the master regulators of B cell development, and differentiation into plasma cells might be of pivotal importance in the generation of autoantibodies in RA, which has been already described in SLE (76) and some myopathies (77). Further mechanistic studies are needed to confirm such association and describe the detailed mechanism.

This article provides evidence of the limited use of the gene expression of these markers to differentiate the early stages of RA, although *MXA, MXB*, and *IFI6* showed an increased expression when compared to HC. The participation of several genes of the IFN signature is also described in the context of the available data on the pathogenesis of the disease. We describe a plausible link between type I IFN-induced gene expression and the generation of anti-CarP antibodies and ACPA. Limitations of the study include sample size and previous DMARD use in the cRA group.

## Author Contributions

JC-D, YB-H, LE-M, DO-C, DS-A, and AA-N: manuscript, experiment, analysis, and idea conception; CR-R, JC, and PM-T: supervision and sample collection; JE-M: overall supervision, analysis, manuscript approval, and conception of idea.

## Conflict of Interest Statement

The authors declare that the research was conducted in the absence of any commercial or financial relationships that could be construed as a potential conflict of interest.
