# Supplementary material for: Type I Interferon Gene Response Is Increased in Early and Established Rheumatoid Arthritis and Correlates with Autoantibody Production
Source: Front Immunol. 2017 Mar 20;8:285. doi: 10.3389/fimmu.2017.00285 (PMC5357778; doi:10.3389/fimmu.2017.00285)
Supplement: Supplementary file 1 [file Table_1.DOCX]

**Supplementary table1. - The type I interferon signature genes and function**

| **Gene** | **Function** |
| --- | --- |
| **IFIT1** | **Interferon-Induced Protein With Tetratricopeptide Repeats 1,** specifically binds single-stranded RNA bearing a  5'-triphosphate group (PPP-RNA), thereby acting as a sensor of viral single-stranded RNAs and inhibiting  expression of viral messenger RNAs. |
| **IFIT2** | **Interferon-Induced Protein With Tetratricopeptide Repeats 2,** IFN-induced antiviral protein which inhibits expression of viral messenger RNAs lacking 2'-O-methylationof the 5' cap. Binds AU-rich viral RNAs, with or without 5' triphosphorylation, RNA-binding isrequired for antiviral activity. Can promote apoptosis |
| **IFI44L** | **Interferon-Induced Protein 44-Like, unknown** |
| **ISG15** | **ISG15 Ubiquitin-Like Modifier,** Ubiquitin-like protein which plays a key role in the innate immune response to viral infection eithervia its conjugation to a target protein (ISGylation) or via its action as a free or unconjugated protein.  ISGylation involves a cascade of enzymatic reactions involving E1, E2, and E3 enzymes which catalyze theconjugation of **ISG15** to a lysine residue in the target protein. Its target proteins include IFIT1, MX1/MxA,PPM1B, UBE2L6, UBA7, CHMP5, CHMP2A, CHMP4B and CHMP6. Can also isgylate: EIF2AK2/PKR which results in its activation. The secreted form of **ISG15** can: induce natural killer cell proliferation,act as a chemotactic factor for neutrophils and act as an inducer ofIFN-gamma. |
| **MXA** | **Myxovirus (Influenza Virus) Resistance 1, Interferon-Inducible ProteinP78,**  Interferon-induced dynamin-like GTPase with antiviral activity against a wide range of RNA viruses andsome DNA viruses. Enhances ER stress-mediated cell death. May regulate the calcium channel activity of TRPCs |
| **MXB** | **Myxovirus (Influenza Virus) Resistance 2,** Interferon-induced dynamin-like GTPase with potent antiviral activity against human immunodeficiencyvirus type 1 (HIV-1). May play a role in regulating nucleocytoplasmictransport and cell-cycle progression |
| **EPSTRLI1** | Unknown, associated with epithelial mesenchymetransition |
| **RSAD** | **Radical S-Adenosyl Methionine Domain Containing 2.** Interferon-inducible iron-sulfur (4FE-4S) cluster-binding antiviral protein that plays a major role inthe cell antiviral state induced by type I and type II interferon. Can inhibit a wide range of DNA and RNAviruses, including human cytomegalovirus (HCMV), hepatitis C virus (HCV), west Nile virus (WNV), dengue virus,sindbis virus, influenza A virus, sendai virus, vesicular stomatitis virus (VSV), and human immunodeficiencyvirus (HIV-1). Displays antiviral activity against influenza A virus by inhibiting the budding of the virus fromthe plasma membrane by disturbing the lipid rafts. This is accomplished, at least in part, through binding andinhibition of the enzyme farnesyldiphospate synthase (FPPS), which is essential for the biosynthesis ofisoprenoid-derived lipids. Promotes TLR7 and TLR9-dependent production of IFN-beta production in plasmacytoiddendritic cells (pDCs) by facilitating Lys-63'-linked ubiquitination of IRAK1. Plays a role in CD4+ T-cells  activation and differentiation. Facilitates T-cell receptor (TCR)-mediated GATA3 activation and optimal T helper  2 (Th2) cytokine production by modulating NFKB1 and JUNB activities. Can inhibit secretion of soluble proteins |
| **HERC5** | Major E3 ligase for ISG15 conjugation. Acts as a positive regulator of innate antiviral response in  cells induced by interferon. Functions as part of the ISGylation machinery that recognizes target proteins in a  broad and relatively non-specific manner. Catalyzes ISGylation of IRF3 which results in sustained activation, it  attenuates IRF3-PIN1 interaction, which antagonizes IRF3 ubiquitination and degradation, and boosts the antiviral  response. Catalyzes ISGylation of influenza A viral NS1 which attenuates virulence; ISGylated NS1 fails to form  homodimers and thus to interact with its RNA targets. Catalyzes ISGylation of papillomavirus type 16 L1 protein  which results in dominant-negative effect on virus infectivity. Physically associated with polyribosomes, broadly  modifies newly synthesized proteins in a cotranslational manner. In an interferon-stimulated cell, newly  translated viral proteins are primary targets of ISG15 |
| **LY6E** | Lymphocyte Antigen 6 Complex, Locus E, LY6E (lymphocyte antigen 6 complex, locus E) is a protein-coding gene. Diseases associated with LY6E include [subvalvular aortic stenosis](http://www.malacards.org/card/subvalvular_aortic_stenosis), and [acute promyelocytic leukemia](http://www.malacards.org/card/acute_promyelocytic_leukemia). |
| **IFI6** | **Interferon, Alpha-Inducible Protein 6**  This gene was first identified as one of the many genes induced by interferon. The encoded protein may play a  critical role in the regulation of apoptosis. A minisatellite that consists of 26 repeats of a 12 nucleotide  repeating element resembling the mammalian splice donor consensus sequence begins near the end of the second  exon. Alternatively spliced transcript variants that encode different isoforms by using the two downstream repeat  units as splice donor sites have been described. (provided by RefSeq, Jul 2008) |
| **IFI35** | **Interferon-induced 35 kDa protein,** |
